# Supplementary material for: Structural basis for Epstein–Barr virus host cell tropism mediated by gp42 and gHgL entry glycoproteins
Source: Nat Commun. 2016 Dec 8;7:13557. doi: 10.1038/ncomms13557 (PMC5155155; doi:10.1038/ncomms13557)
Supplement: Supplementary Information — Supplementary References 1-9 and Supplementary Tables 1-3 [file ncomms13557-s1.pdf]

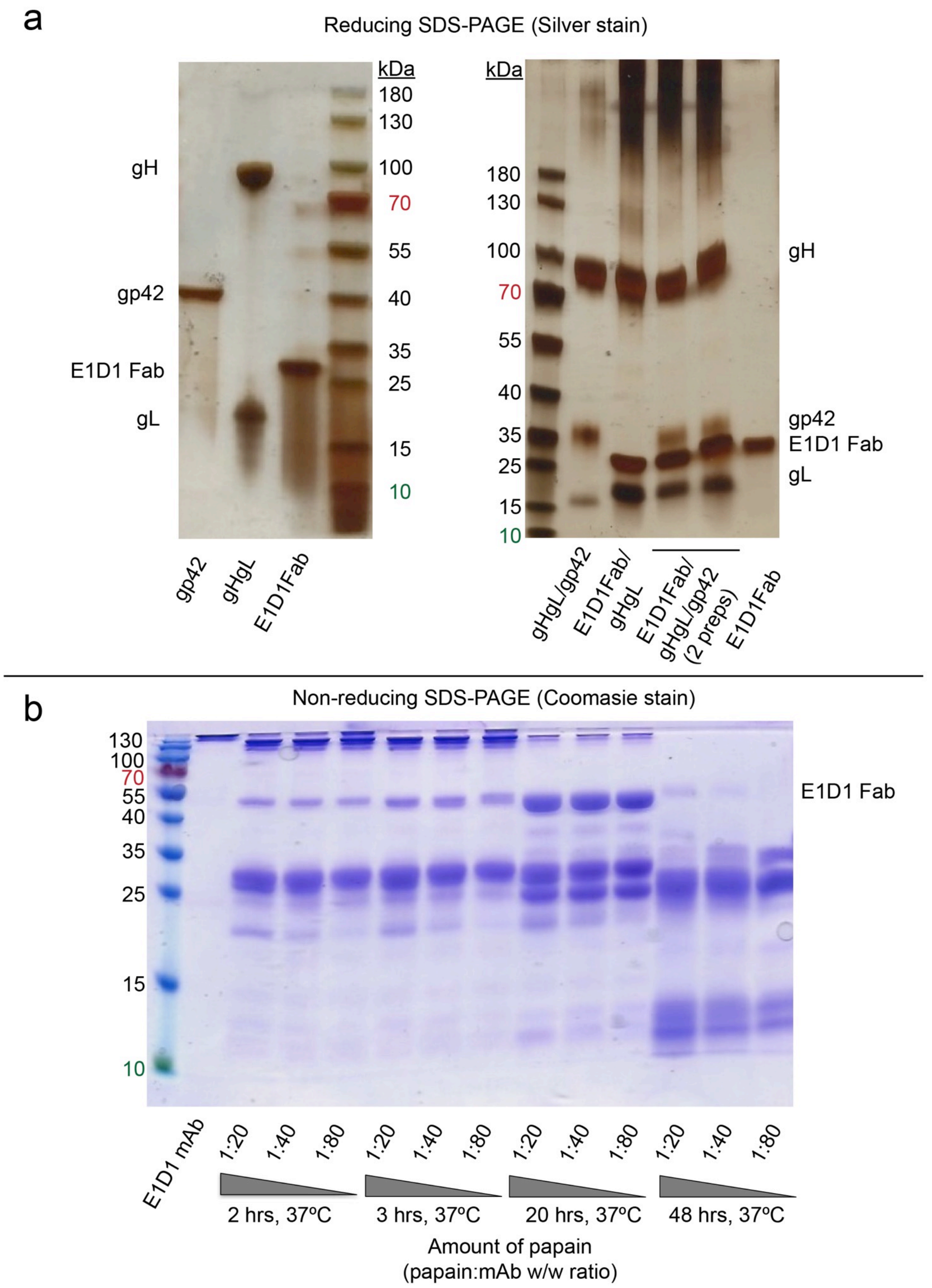


**Supplementary Figure S1. Characterization of gHgL/gp42/E1D1 complex and generation of 50 kDa Fab fragment by controlled enzymatic digestion of E1D1.** (**a**) SDS-PAGE analysis under reducing conditions (silver-stain) of gHgL, gp42, and E1D1 Fab complexes. (**b**) SDS-PAGE analysis under non-reducing conditions (coomasie stain) for generation of E1D1 Fab from intact E1D1.

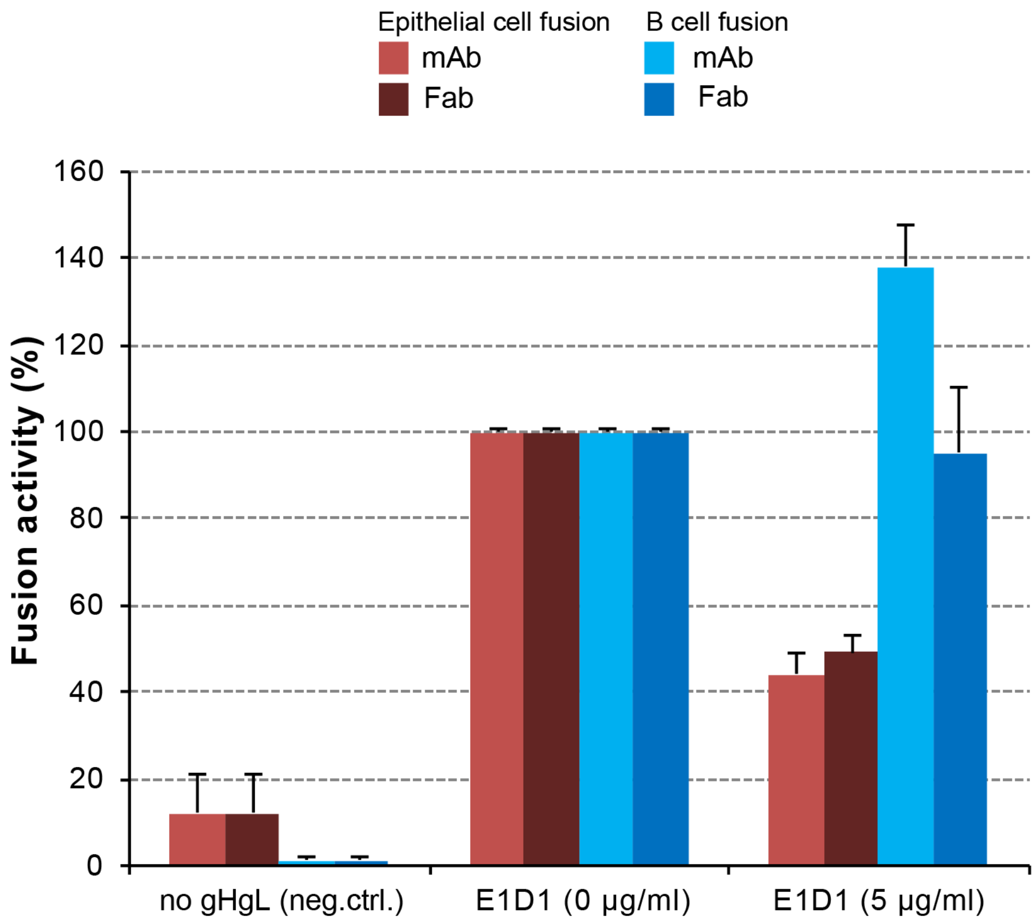


**Supplementary Figure S2. Partial inhibition of epithelial cell fusion activity by E1D1 Fab and mAb**. Epithelial cell fusion activity is represented as the average of three biological replicates and shown as maroon histogram bars. B cell fusion activity is similarly shown as blue bars. Lighter shades denote addition of mAb and darker shade denote Fab addition during the fusion assay. Error bars are ±S.D. (Standard Deviation).

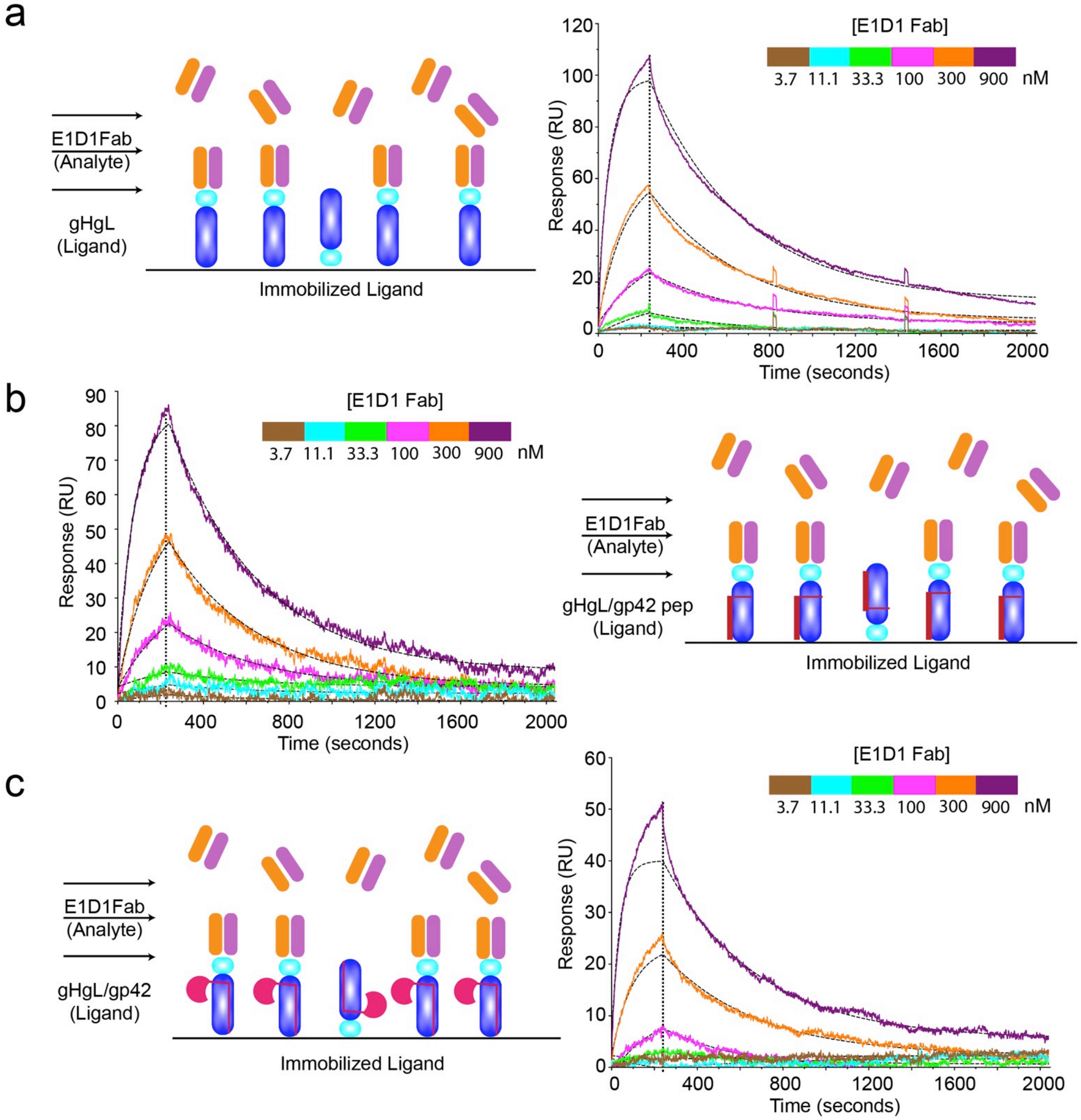


**Supplementary Figure S3. E1D1 affinity for gHgL is not affected by gp42 or gp42-peptide.** Surface Plasmon Resonance (SPR) measurements of E1D1 Fab binding to (**a**) gHgL, (**b**) gHgL/N-domain peptide and (**c**) gHgL/gp42. gHgL, gHgL/gp42pep, or gHgL/gp42 was used as the immobilized ligand while E1D1 Fab was used as the soluble analyte (mobile phase). The kinetics data (on-rate and off-rate) and affinity ($K_D$) values from a global fit of a 1:1 interaction model are collected in Table 1.

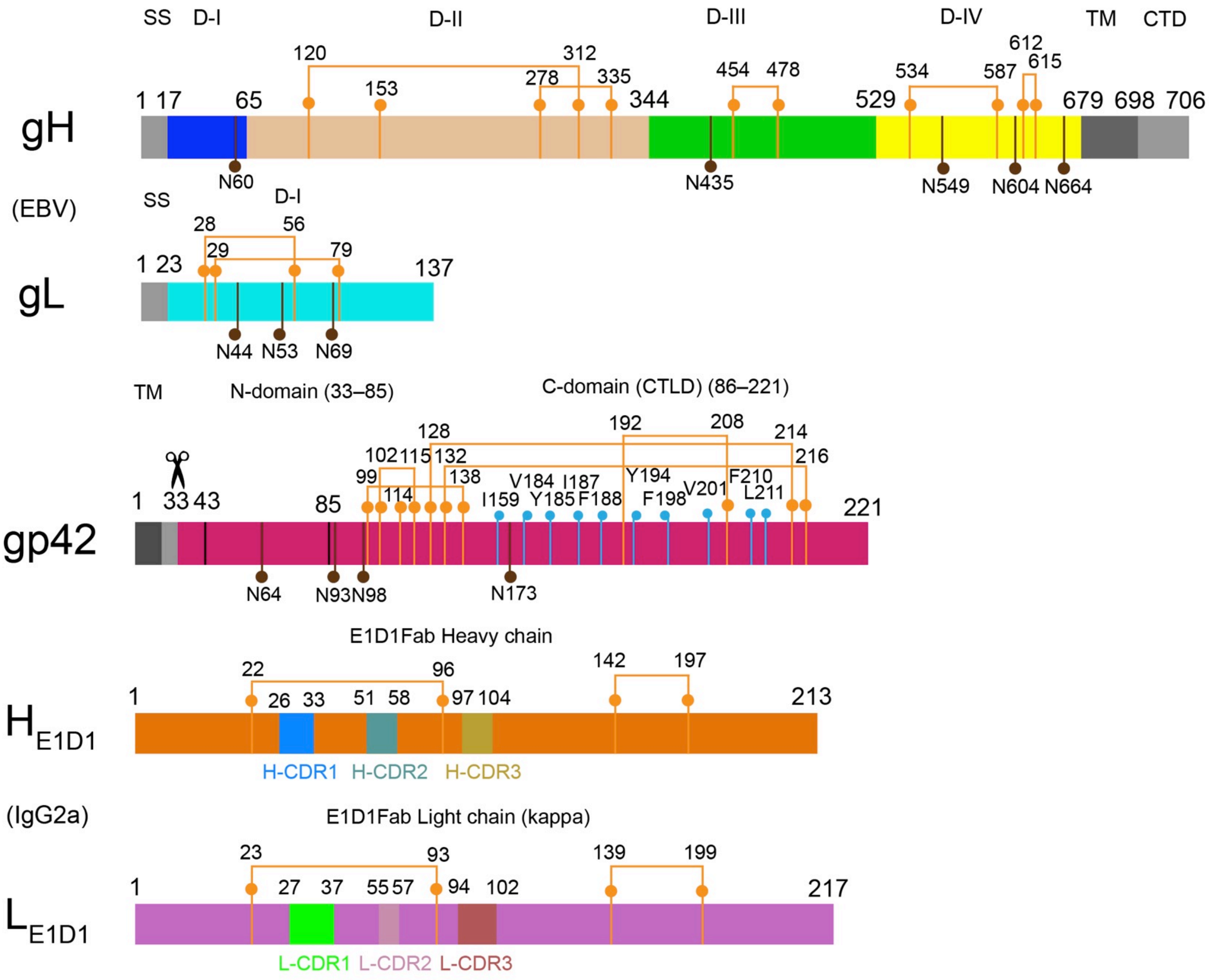


**Supplementary Figure S4. Schematic representations of gHgL, gp42 and E1D1 Fab proteins.** The 2-D bars represent the domain boundaries of gH, gL, gp42 and E1D1 Fab. The bar diagrams of individual domains are colored using the same coloring scheme as in Fig. 2. Potential N-linked glycosylation sites are labeled and numbered by downward pointing brown 'lollipops' and cysteines are numbered and shown as upward pointing orange 'lollipops'. Orange connected horizontal lines show disulfide bridges. C153 in gH D-II and C114 in gp42 are unpaired cysteines. The cleavage site for the gp42 N-terminal anchor is shown by a scissor. SS stands for signal sequence, TM for transmembrane and CTD for C-terminal domain.

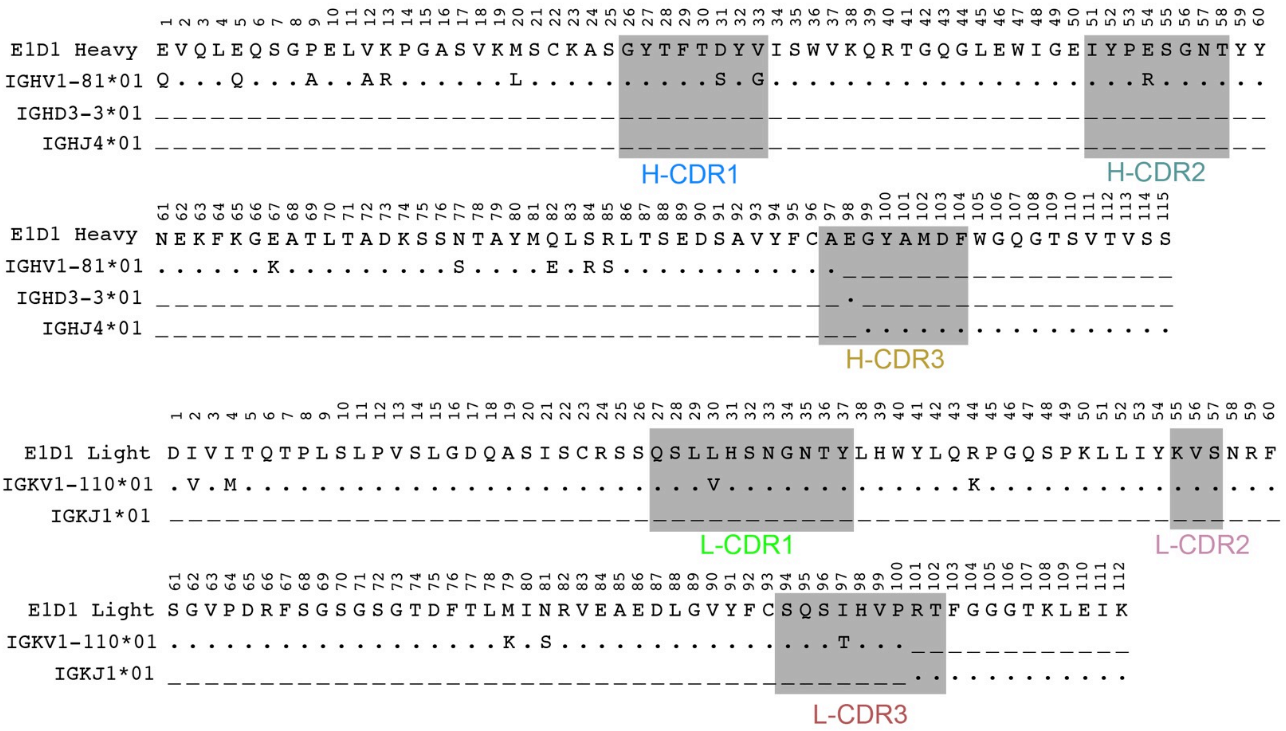


**Supplementary Figure S5. Sequences of the E1D1 $V_H$ and $V_L$ domains.** The amino acid sequence of E1D1 heavy and light chain was obtained using cDNA isolated from E1D1 expressing hybridoma cells. The alignment shows the closest V-D-J allelic regions for the heavy and V-J regions for the light chain (imgt.org/IMGT_vquest) giving the inferred unmutated common ancestor (UCA) sequence. Dot '.' denotes identity and dash '-' denotes a gap. Residues that differ due to presumed somatic mutation are represented by the original amino acid single letter code in the alignment. Grey box background highlights the E1D1 Complementarity Determining Regions (CDRs). The heavy chain CDRs are labeled H-CDR1 through 3 and the light chain CDRs are labeled L-CDR1 through 3. Coloring scheme for the CDR labels is identical to the coloring for those regions in the structure as represented in Fig. 5 and in Supplementary Fig. S4.

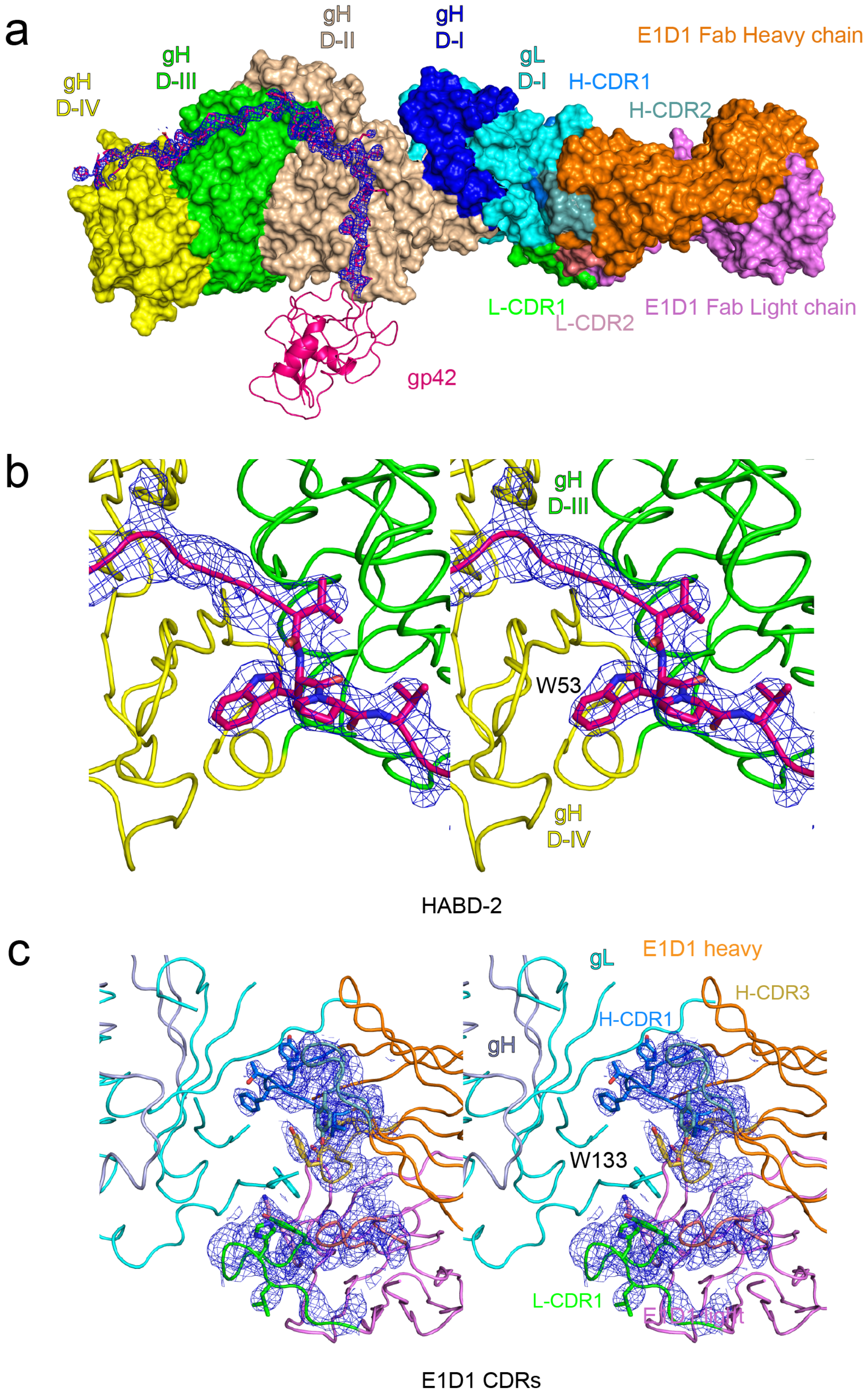


**Supplementary Figure S6. Examples of electron density observed for gp42 and E1D1 model building.** Simulated annealing (SA) composite omit maps contoured at 1.0 σ for (**a**) gp42 N-domain (43–85), (**b**) High Affinity Binding Determinant 2 (HABD-2) as from Fig. 3c, and (**c**) E1D1 CDR's showing the correspondence with the modeled amino acids in those spatial positions as in the crystal structure. Panels (**b**) and (**c**) are shown as stereo pairs.

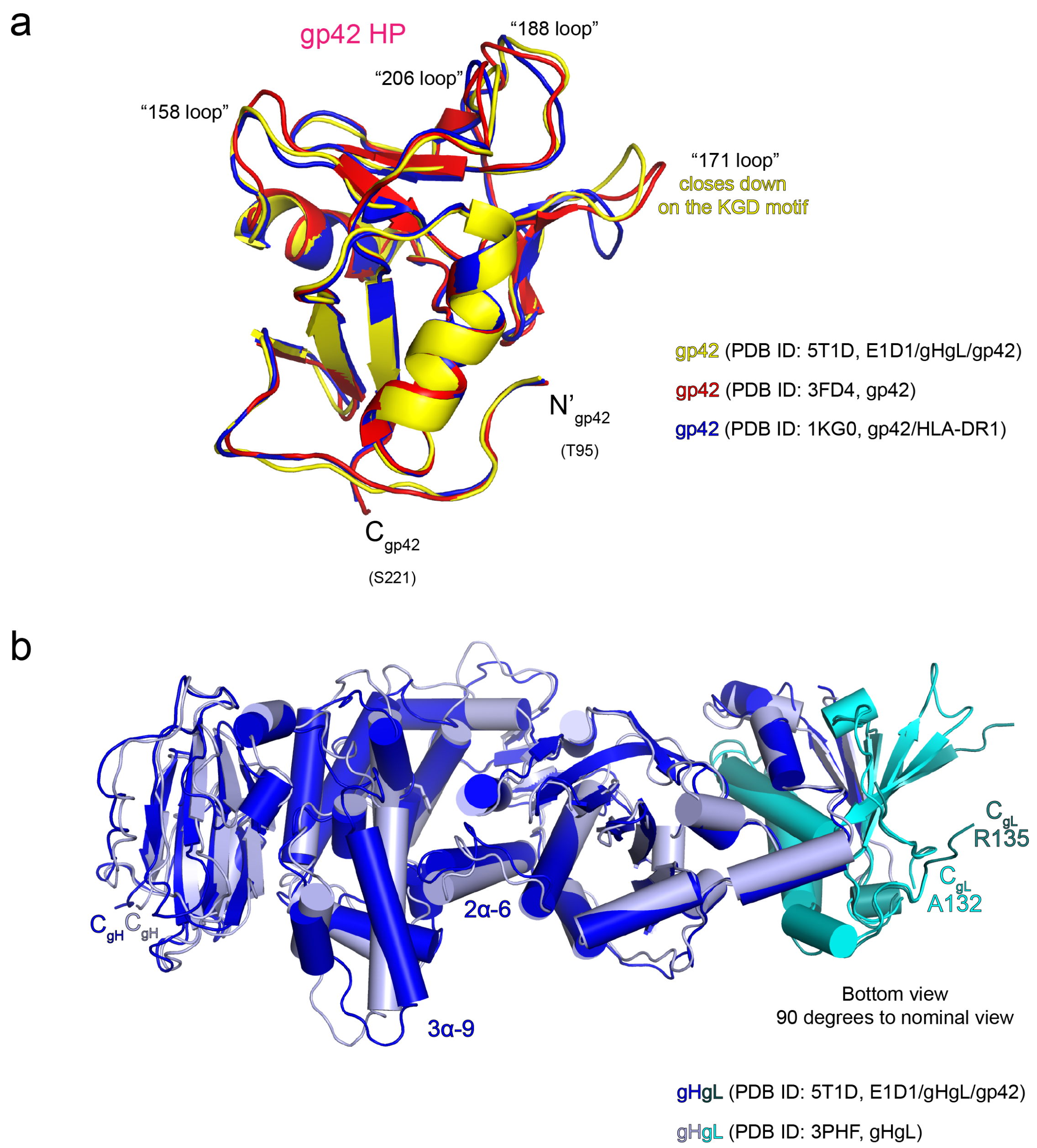


**Supplementary Figure S7. Structural differences in EBV gHgL and gp42 in known crystal structures.** Structural alignments for (**a**) gp42, and (**b**) gHgL from different crystal structures highlighting notable differences. Legends to denote the coloring of different domains is included in each panel within the figure.

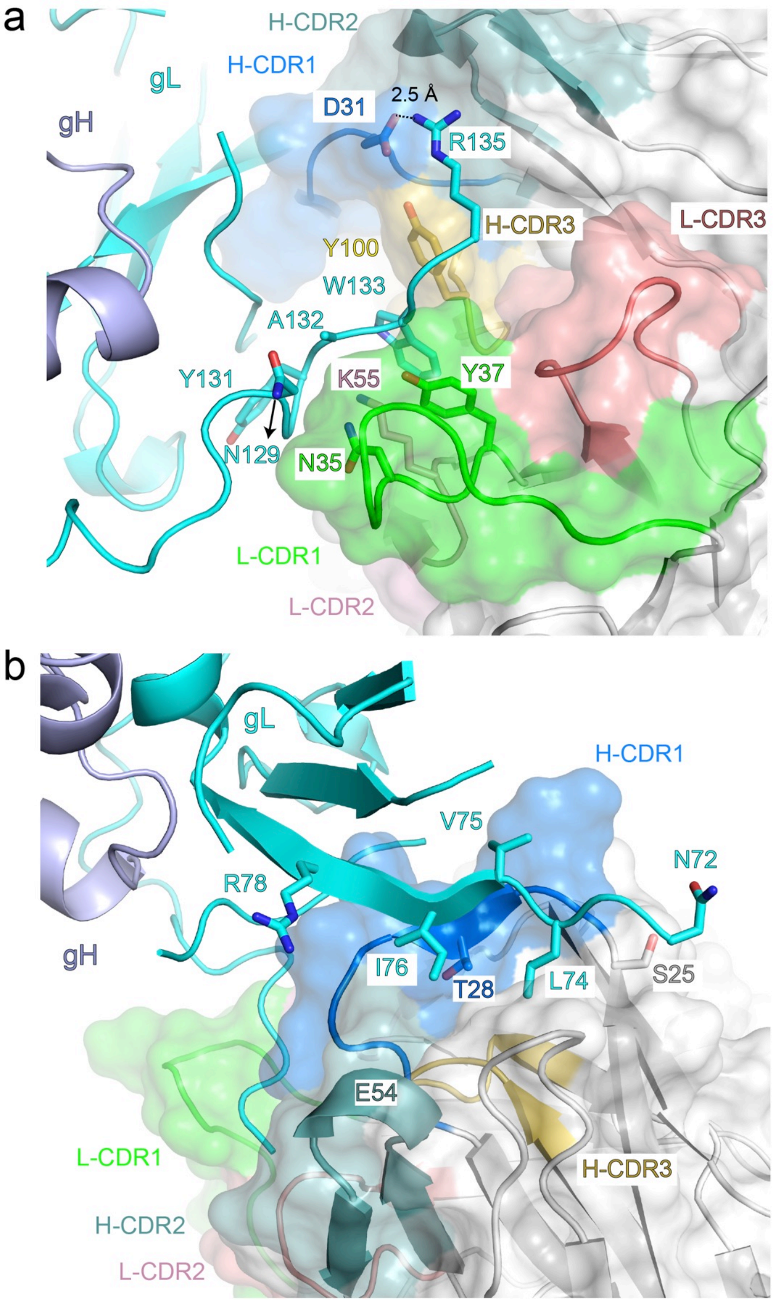


**Supplementary Figure S8. Interactions of E1D1 residues with gL.** (**a**) Interactions between gL and E1D1 residues shown in stick format. (**b**) Interactions between gL β-3 strand (Lβ-3, residues 72–79) and E1D1 heavy chain CDR. gL:L74–I76 is buried in a shallow hydrophobic cleft within H-CDR1. For all panels H-CDR1 is colored marine, H-CDR2 is lightteal, H-CDR3 is sulfur; and, L-CDR1 is green, L-CDR2 is pink, and L-CDR3 is deepsalmon (coloring scheme label as from Pymol). gH is colored lightblue and gL in cyan. Structures were rendered using MacPyMol.

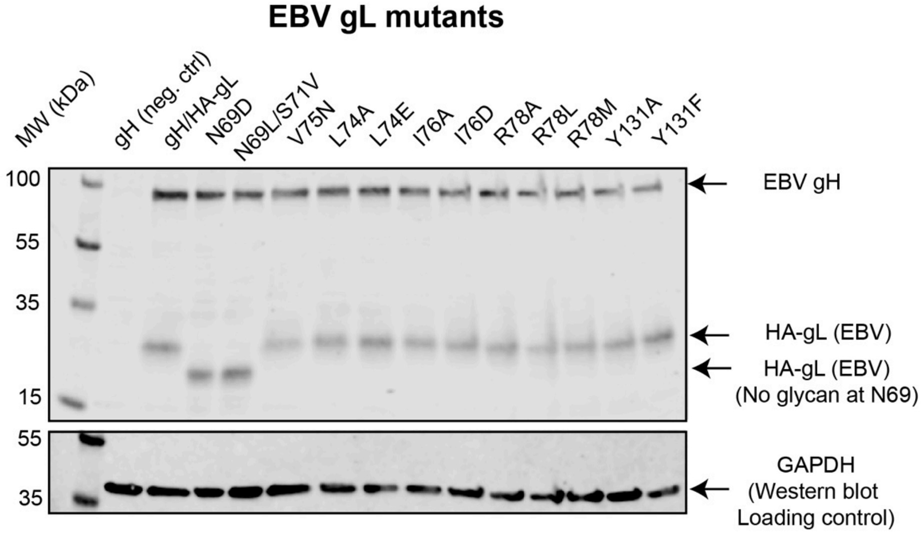


**Supplementary Figure S9. Expression levels of different gL mutants.** Western blot of gHgL mutants detected using a polyclonal anti-gHgL antibody (rabbit). GAPDH bands are shown as a loading control and shows similar levels of expression of all mutants tested in the cell-cell fusion assays (Fig. 6).

**Supplementary Table S1. EBV gH residue contacts with gp42 N-domain**. Gp42 residues (43–85) are arranged in descending order of the most buried, non-zero difference in Accessible Surface Area (ASA) as calculated by naccess, 76 gH residues contact gp42 N-domain.

| Structural feature (gH subsite) | gH residue | Buried surface area (Change in ASA) ($Å^2$) | Nearby gp42 residues |
|---|---|---|---|
| HABD-4 | Y132 | 107.91 | E68, Y71, G72, D73 |
| Linker | R350 | 102.18 | F63, N64, K65 |
| HABD-2 | E362 | 87.16 | V52 |
| HABD-4 | Y346 | 79.23 | A67, E70, Y71 |
| HABD-4 | Y133 | 76.29 | Y71, D73, V76 |
| Turn between HABD-1–3 and HABD-4–5 | I134 | 75.27 | K74, E75 |
| HABD-1 | I613 | 72.60 | W44, P46 |
| HABD-3 | Y389 | 63.98 | P57, P58, P59 |
| HABD-2 | I548 | 62.35 | W53 |
| HABD-5 | Y157 | 51.56 | V76, L78, P79 |
| HABD-5 | T170 | 50.59 | L78, P79, W81 |
| HABD-1 | C615 | 48.68 | K47, P48, N49 |
| HABD-5 | Q168 | 47.85 | W81 |
| HABD-2 | N442 | 45.91 | V52, W53, P54 |
| HABD-5 | D165 | 44.75 | W81, T82, T84 |
| HABD-4 | Q344 | 42.80 | Y71 |
| HABD-2 | L401 | 39.58 | N49, V50, E51 |
| HABD-2 | P441 | 38.63 | W53 |
| HABD-2 | T635 | 36.37 | N49, V50, E51 |
| HABD-5 | V185 | 35.09 | P83 |
| HABD-1 | F617 | 34.01 | K47 |
| HABD-1 | C612 | 32.45 | P46, K47 |
| Linker | P109 | 31.37 | F63 |
| HABD-2 | L445 | 31.17 | W53 |
| HABD-5 | T136 | 26.37 | K77 |

| | | | |
|---|---|---|---|
| HABD-3 | Y383 | 25.65 | P59, P60 |
| Linker | M354 | 25.41 | F63 |
| HABD-3 | V388 | 24.31 | P59, P60 |
| D-I/D-II groove | R152 | 22.90 | E75 |
| HABD-5 | E187 | 22.54 | L85, H86 |
| HABD-3 | A357 | 22.52 | P58, P59 |
| HABD-5 | S161 | 20.63 | W81 |
| HABD-2 | S636 | 19.74 | N49 |
| HABD-3 | A353 | 19.60 | P60, V61 |
| HABD-1 | S611 | 17.95 | P46 |
| HABD-5 | A159 | 17.94 | P79 |
| HABD-3 | Q439 | 17.92 | V55 |
| HABD-5 | G135 | 17.57 | V76, K77 |
| HABD-5 | K341 | 17.50 | V76 |
| HABD-1 | I602 | 16.96 | W44 |
| HABD-5 | K166 | 16.23 | T84, L85, H86 |
| HABD-1 | F614 | 15.69 | W44 |
| HABD-5 | G164 | 14.25 | T84 |
| HABD-3 | G391 | 14.09 | V55 |
| HABD-5 | L155 | 13.77 | K77, L78 |
| Linker | V107 | 12.52 | F63 |
| HABD-1 | T663 | 11.32 | K47, P48 |
| HABD-5 | A172 | 10.87 | L78 |
| HABD-1 | M668 | 10.39 | W44 |
| HABD-1 | Q609 | 9.45 | W44 |
| HABD-1 | F605 | 8.85 | W44 |
| HABD-1 | G616 | 8.52 | K47 |
| HABD-5 | L160 | 8.14 | W81 |

| | | | |
|---|---|---|---|
| HABD-4 | Q129 | 8.04 | Y71 |
| HABD-2 | Q637 | 7.67 | N49 |
| HABD-3 | M361 | 7.01 | D56, P57, P58 |
| HABD-2 | G394 | 7.01 | V55 |
| HABD-2 | S398 | 6.20 | V52 |
| HABD-1 | N604 | 5.44 | T43, W44 |
| HABD-2 | T397 | 5.31 | V52 |
| HABD-5, KGD Motif | G189 | 5.12 | L85 |
| HABD-3 | M356 | 4.95 | P58 |
| HABD-4/HABD-5 | H154 | 4.93 | K74 |
| HABD-2 | Y633 | 4.23 | V50 |
| HABD-1 | T662 | 4.04 | W44 |
| HABD-5 | M137 | 3.83 | L78 |
| HABD-1 | L660 | 3.26 | W44 |
| HABD-4 | E349 | 2.69 | E70, Y71 |
| HABD-5 | P139 | 2.59 | L78 |
| HABD-2 | E638 | 1.72 | N49 |
| Linker | H108 | 0.92 | F63 |
| HABD-5, KGD Motif | K188 | 0.70 | L85, H86 |
| HABD-5 | Y169 | 0.63 | W81 |
| HABD-5 | K183 | 0.36 | W81, T82 |
| HABD-5 | T186 | 0.32 | L85 |
| HABD-2 | N549 | 0.18 | W53 |

**Supplementary Table S2. Interactions in each of the 5 EBV gH high affinity binding determinants (HABD) with gp42.**

| Structural feature (gH subsite) | gH residue/atom | gp42 residue/atom | Interaction type | Distance (Å) |
|---|---|---|---|---|
| HABD-1 | F605/CD1 | W44/CD1 | Van der Waals | 5.6 |
| | F614/CE1 | W44/CD1 | Van der Waals | 6.2 |
| | I613/CD1 | W44/CG | Van der Waals | 3.4 |
| | F614/CE1 | W44/CZ3 | Van der Waals | 4.1 |
| | I602/CD1 | W44/CZ2 | Van der Waals | 4.7 |
| | L660/CD2 | W44/CH2 | Van der Waals | 4.4 |
| | C612/O | K47/N | H-bond | 2.8 |
| HABD-2 | I548/CG2 | W53/CH2 | Van der Waals | 4.0 |
| | I548/CG1 | W53/CE2 | Van der Waals | 5.6 |
| | L445/CD2 | W53/CD1 | Van der Waals | 5.0 |
| | P441/CB | W53/CD1 | Van der Waals | 3.8 |
| | N442/OD1 | W53/N | H-bond | 3.0 |
| | N442/ND2 | W53/O | H-bond | 2.9 |
| | L445/CD2 | V52/CG2 | Van der Waals | 4.8 |
| | L401/CD2 | V52/CG2 | Van der Waals | 4.1 |
| | T397/CG2 | V52/CG2 | Van der Waals | 4.5 |
| | G394/CA | V52/CG1 | Van der Waals | 4.0 |
| | E362/CB | V52/CB | Van der Waals | 4.6 |
| HABD-3 | Y389/CE1 | P58/CG | Van der Waals | 4.0 |
| | Y389/CG | P59/CD | Van der Waals | 3.9 |
| | G391/CA | V55/CG2 | Van der Waals | 4.4 |
| | Y383/CE1 | P59/CG | Van der Waals | 4.8 |
| HABD-4 | Q344/CG | Y71/CE1 | Van der Waals | 4.3 |
| | Y346/CE2 | Y71/CZ | Van der Waals | 4.4 |
| | Q129/CG | Y71/CE2 | Van der Waals | 4.9 |
| | Y346/N | Y71/OH | H-bond | 3.0 |
| | Y132/CG | Y71/CD2 | Van der Waals | 3.5 |
| | Y133/OH | G72/N | H-bond | 3.7 |
| HABD-5 | Y157/CD2 | L78/CD1 | Van der Waals | 4.1 |
| | A172/CB | L78/CD2 | Van der Waals | 4.0 |
| | T170/CG2 | L78/CD2 | Van der Waals | 4.4 |
| | A159/CB | P79/CG | Van der Waals | 3.7 |
| | S161/OG | W81/NE1 | H-bond | 3.4 |
| | Q168/NE2 | W81/O | H-bond | 3.7 |
| | Q168/CB | W81/CZ3 | Van der Waals | 3.8 |

**Supplementary Table S3. Cell-cell fusion assay experimental data for Figure 6.**

| EBV gL mutant | Surface Expression (anti-HA) | | | Epithelial cell fusion | | | B cell fusion | | |
|---|---|---|---|---|---|---|---|---|---|
| | Mean | ±S.D. | N | Mean | ±S.D. | N | Mean | ±S.D. | N |
| gH (neg. ctrl) | 12 | 3 | 3 | 12 | 8 | 3 | 1 | 0 | 3 |
| gH/HA-gL | 100 | 4 | 3 | 100 | 4 | 3 | 100 | 5 | 3 |
| N69D | 104 | 13 | 3 | 106 | 10 | 3 | 89 | 6 | 3 |
| N69L | 99 | 1 | 3 | 224 | 40 | 3 | 113 | 13 | 3 |
| S71V | 104 | 6 | 3 | 272 | 26 | 3 | 116 | 15 | 3 |
| N69L/S71V | 95 | 10 | 3 | 279 | 70 | 3 | 93 | 9 | 3 |
| L74A | 101 | 8 | 3 | 71 | 11 | 3 | 85 | 15 | 3 |
| L74E | 97 | 7 | 3 | 46 | 11 | 3 | 97 | 9 | 3 |
| V75N | 99 | 7 | 3 | 71 | 12 | 3 | 90 | 11 | 3 |
| I76A | 98 | 10 | 3 | 72 | 10 | 3 | 94 | 7 | 3 |
| I76D | 99 | 5 | 3 | 70 | 13 | 3 | 80 | 8 | 3 |
| R78A | 91 | 8 | 3 | 71 | 9 | 3 | 84 | 6 | 3 |
| R78L | 90 | 10 | 3 | 52 | 8 | 3 | 64 | 9 | 3 |
| R78M | 101 | 11 | 3 | 51 | 6 | 3 | 88 | 13 | 3 |
| L74A/I76A/R78A | 99 | 5 | 3 | 42 | 11 | 3 | 90 | 11 | 3 |
| Y131A | 103 | 11 | 3 | 45 | 9 | 3 | 95 | 13 | 3 |
| Y131F | 100 | 8 | 3 | 119 | 5 | 3 | 79 | 15 | 3 |

S.D. is Standard Deviation, N is Number of biological replicates, neg. ctrl is negative control which here is gH vector alone without gL
